# Racial disparities and utilization trends of first-line targeted therapies for metastatic breast cancer

**DOI:** 10.1093/jncics/pkag043

**Published:** 2026-04-24

**Authors:** Yehoda M Martei, Kan Chen, Modesty Obasohan, Ronac Mamtani, Erin Aakhus, Lawrence N Shulman, Rebecca A Hubbard, Amy Clark

**Affiliations:** Division of Hematology and Oncology, Department of Medicine, University of Pennsylvania, Philadelphia, PA, United States; Abramson Cancer Center, University of Pennsylvania, Philadelphia, PA, United States; Department of Biostatistics, Harvard University, Boston, MA, United States; Perelman School of Medicine, University of Pennsylvania, Philadelphia, PA, United States; Division of Hematology and Oncology, Department of Medicine, University of Pennsylvania, Philadelphia, PA, United States; Abramson Cancer Center, University of Pennsylvania, Philadelphia, PA, United States; Division of Hematology and Oncology, Department of Medicine, University of Pennsylvania, Philadelphia, PA, United States; Abramson Cancer Center, University of Pennsylvania, Philadelphia, PA, United States; Division of Hematology and Oncology, Department of Medicine, University of Pennsylvania, Philadelphia, PA, United States; Abramson Cancer Center, University of Pennsylvania, Philadelphia, PA, United States; Department of Biostatistics, Epidemiology, and Informatics, University of Pennsylvania, Philadelphia, PA, United States; Department of Biostatistics, Brown University, Providence, RI, United States; Division of Hematology and Oncology, Department of Medicine, University of Pennsylvania, Philadelphia, PA, United States; Abramson Cancer Center, University of Pennsylvania, Philadelphia, PA, United States

## Abstract

**Background:**

We aimed to determine temporal trends and racial disparities in utilization and time to treatment initiation of cyclin dependent kinase 4/6 (CDK4/6) inhibitors and pertuzumab for first-line metastatic breast cancer.

**Methods:**

We extracted data from a nationwide electronic health record–derived deidentified database. Female patients aged 18 years and older with estrogen receptor–positive and HER2-negative or HER2-positive metastatic breast cancer eligible for CDK4/6 inhibitors (March 2015-October 2021) or pertuzumab (July 2012-September 2021) were included. Our outcomes were adjusted temporal trends in the proportion of patients receiving respective therapies using logistic regression with natural cubic splines for time trends and tested for changes in utilization over time within and between racial groups (non-Hispanic White or non-Hispanic Black). Similar models using linear regression estimated mean time to treatment initiation.

**Results:**

A total of 5173 (non-Hispanic White = 4478; non-Hispanic Black = 695) estrogen receptor–positive and HER2-negative and 2321 (non-Hispanic White = 1915; non-Hispanic Black = 406) HER2-positive metastatic breast cancer patients were included. There were statistically significant differences in the proportion initiating CDK4/6 inhibitors over time within racial groups (non-Hispanic White: 23.5%, 95% confidence interval [CI] = 20.1% to 27.3%, in 2015 to 53.8%, 95% CI = 48.6% to 59.0%, in 2021; non-Hispanic Black: 20.6%, 95% CI = 11.9% to 33.0%, in 2015 to 73.6%, 95% CI = 61.7% to 83.0%, in 2021) and between groups (*P* = .009). There was a statistically significant increase in utilization of pertuzumab within both racial groups over time (*P* < .001), but no statistically significant difference between groups (*P* = .45). Time to treatment initiation decreased over time with no statistically significant differences in time to treatment initiation trends between the 2 groups.

**Conclusions:**

Utilization of targeted therapies increased over time, however non-Hispanic Black patients were less likely to receive CDK4/6 inhibitors compared with non-Hispanic White. Approximately half of eligible patients did not receive pertuzumab. Further research is needed to understand mediators and design interventions to address underutilization of these therapies and those contributing to racial disparities in CDK4/6 inhibitor utilization.

## Introduction

Breast cancer mortality has declined in the past 40 years, partly attributable to novel therapies for breast cancer. However, non-Hispanic Black women continue to have the highest standardized breast cancer mortality rate of any ethnic or racial group in the United States.^1^^,^^2^ Similar trends have been reported across different breast cancer receptor subtypes, including hormone receptor–positive and HER2–positive disease.^3-5^ Although the reasons for the persistent gap in mortality are complex, data point to disparities along the cancer care continuum that contribute to differences in outcomes experienced by racial and ethnic minorities, including delays in multimodality therapy initiation and undertreatment.^6-9^ For instance, non-Hispanic Black women were more likely than non-Hispanic White women to have at least 60-day delay in initiating treatment following a breast cancer diagnosis across all socioeconomic status levels.^7^ Additionally, data showed that non-Hispanic Black women were more likely than non-Hispanic White women to have adjuvant trastuzumab omitted when indicated (adjusted odds ratio [OR] = 3.14, 95% confidence interval [CI] = 1.38 to 7.17).^10^ Furthermore, non-Hispanic Black women with hormone receptor–positive and HER2-negative breast cancer were less likely to receive guideline-concordant care compared with non-Hispanic White women.^11^

Recent US Food and Drug Administration (FDA) approvals of oral CDK4/6 inhibitors^12-14^ and intravenous (IV) pertuzumab^15^ in the first-line setting for metastatic breast cancer have the potential to further improve progression-free survival and overall survival among patients with breast cancer. However, less is known about the overall utilization of these medicines in the United States, including racial and ethnic disparities in utilization of these targeted therapies. Pertuzumab received FDA accelerated approval for first-line metastatic breast cancer in 2012, and long-term follow-up from the CLEOPATRA study showed improved median overall survival from 40 to 56 months with addition of pertuzumab.^16^ Palbociclib was the first CDK4/6 inhibitor approved in this class of targeted therapy by the FDA in February 2015.^17^ Subsequent trials have shown similar efficacy of other CDK4/6 inhibitors—ribociclib and abemaciclib—which are approved in conjunction with endocrine therapy for first-line hormone receptor–positive and HER2-negative metastatic breast cancer.^14^^,^^18^^,^^19^

Medical insurance reimbursement for IV and oral systemic therapies differs within the United States: IV infusions are covered as a medical benefit, and oral medications, including oral cancer medicines, are covered through a prescription plan that require up-front out-of-pocket copayment.^20^ This variation in reimbursement is likely to disproportionately affect low-income patients and the underinsured and potentially exacerbate preexisting racial disparities in utilization of oral cancer therapies.

As targeted cancer therapies become more widely available, it is critical to understand temporal trends and racial disparities in uptake of these life-saving therapies. In this study, we examined trends in utilization of CDK4/6 inhibitors and pertuzumab as illustrative examples of oral- and IV-targeted therapies. Our objective was to assess overall utilization and whether there were racial disparities in utilization of CDK4/6 inhibitors and pertuzumab for first-line metastatic breast cancer treatment. We hypothesized that non-Hispanic Black women will have lower utilization of the targeted therapies, with disparities more amplified for oral CDK4/6 inhibitors. Second, we evaluated racial disparities in time to treatment initiation for both therapies.

## Methods

### Database and study population

We conducted a retrospective cohort analysis using the Flatiron Health electronic health record (EHR)–derived database.^21^^,^^22^ The data are de-identified and subject to obligations to prevent re-identification and protect patient confidentiality. Eligible female patients included those aged 18 years and older with de novo or recurrent metastatic breast cancer and race identified as non-Hispanic White or non-Hispanic Black. Race and ethnicity were designated as self-reported information.

The estrogen receptor–positive and HER2-negative cohort was selected based on estrogen receptor–positive and HER2-negative metastatic breast cancer receptor status on or after March 1, 2015—representing the month after the FDA approval of the first CDK4/6 inhibitor, palbociclib—to October 2021—representing data cutoff. The HER2-positive cohort was selected based on diagnosis with HER2-positive metastatic breast cancer diagnosed on or after July 1, 2012, the month following the FDA approval of pertuzumab, to September 2021, representing data cutoff. During the study period, the de-identified data originated from approximately 280 US cancer clinics (approximately 800 sites of care).

Our exclusion criteria included presumed incomplete historical treatment data, defined as fewer than 2 documented clinical visits after the index diagnosis or 90 days or more between diagnosis and first subsequent structured activity or treatment initiation. Patients with multiple malignancies, that is, records in multiple cancer cohorts across the set of cancers included in the database, were excluded. The protocol was reviewed by the University of Pennsylvania institutional review board and determined to meet eligibility criteria for institutional review board review exemption.

### Ascertainment of outcomes and covariates

The primary outcome evaluated was a binary indicator of initiation of first-line treatment with any of the 3 CDK4/6 inhibitors (palbociclib, ribociclib, and abemaciclib) for the estrogen receptor–positive and HER2-negative cohort and initiation of pertuzumab for the HER2-positive cohort within 90 days of metastatic breast cancer diagnosis. The secondary outcome of this study was time to treatment initiation defined as the number of days from metastatic breast cancer diagnosis to receipt of either targeted therapy in the first-line setting evaluated among patients initiating targeted therapy within 90 days of metastatic breast cancer diagnosis. Delays in time to treatment initiation have been associated with lower survival among patients with breast cancer.^23^^,^^24^

Other variables included age (assessed as a continuous variable), Eastern Cooperative Oncology Group (ECOG) performance status within 60 days or less prior, to 30 days or more of metastatic breast cancer diagnosis (categorized as 0, 1, 2-4), insurance type (private or commercial; Medicare, Medicaid, and/or other government; or uninsured or self-pay), residence in a state with Medicaid expansion under the Affordable Care Act (ACA) vs not, practice type (academic vs community), and proportion of non-Hispanic Black patients seen in the practice as a continuous variable in 10% increases. Additional clinical variables were metastatic breast cancer site and de novo vs recurrent metastatic breast cancer.

### Statistical analysis

#### Descriptive analysis

We conducted descriptive analyses summarizing the distribution of patient and clinical characteristics stratified by race (non-Hispanic Black vs non-Hispanic White) and by estrogen receptor–positive and HER2-negative vs HER2-positive receptor status. Missing values were imputed using multiple imputation via chained equations, implemented via the MICE package in R. We used 5 imputations and included the following variables: date of metastatic breast cancer diagnosis and age at diagnosis, Medicaid information, insurance type, ECOG performance status, practice type, proportion of Black patients within the practice, race, and utilization of CDK4/6 inhibitors or pertuzumab. We plotted the observed proportion of patients within each cohort initiating first-line treatment with the drug of interest by year of metastatic breast cancer diagnosis stratified by race. We estimated natural cubic splines with 2 degrees of freedom fit to the binary outcome of receipt of any CDK4/6 inhibitors or pertuzumab for the respective cohorts, with exposure variable of continuous time using logistic regression, separately by race.

#### Inferential analysis

We then used logistic regression to estimate adjusted temporal trends in the receipt of CDK4/6 inhibitors and differences in these trends by race. Predictor variables in the models included a natural cubic spline with 2 degrees of freedom for time, race, the interaction between the spline basis functions and race, age at diagnosis, ECOG performance status, insurance status at the time of diagnosis, state Medicaid expansion status (coded as resident in states with Medicaid expansion under ACA vs not at the time of metastatic breast cancer diagnosis), and practice setting (academic vs community practice). Given the potential for variation in the relationship between utilization and race depending on the proportion of Black patients seen at a given hospital,^25^ we assessed the proportion of non-Hispanic Black patients treated at each site within the cohort and included this variable in our regression analysis. Marginal standardization was used to obtain adjusted probabilities of treatment initiation from logistic regression models.

Generalized estimating equations with exchangeable working correlation were used to capture dependence of observations from the same site. We used composite Wald tests of regression coefficients associated with spline basis function main effects (to test the null hypothesis of no change over time in proportion of patients receiving the therapy of interest in non-Hispanic White), regression coefficients associated with spline basis functions and race × spline basis function interaction terms (to test the null hypothesis of no change over time in proportion of patients receiving respective therapy in non-Hispanic Black), and regression coefficients for the race × spline basis function interaction terms alone (to test the null hypothesis of no difference in uptake trends for non-Hispanic White and non-Hispanic Black). A similar model was used for time to treatment initiation, using linear regression models. These analyses were repeated for the HER2-positive metastatic breast cancer cohort using the outcome of first-line treatment with pertuzumab. A 2-sided *P* value less than .05 was considered statistically significant. All statistical analyses were performed using R software (R-4.3.2).

#### Sensitivity analysis

We estimated the proportion of participants who received 2 or more noncancelled CDK4/6 inhibitors or pertuzumab prescription orders. We propose this to potentially separate patients receiving prescriptions written for prior authorization only from patients who truly received the drug.

## Results

### Study population

**Figure 1.**
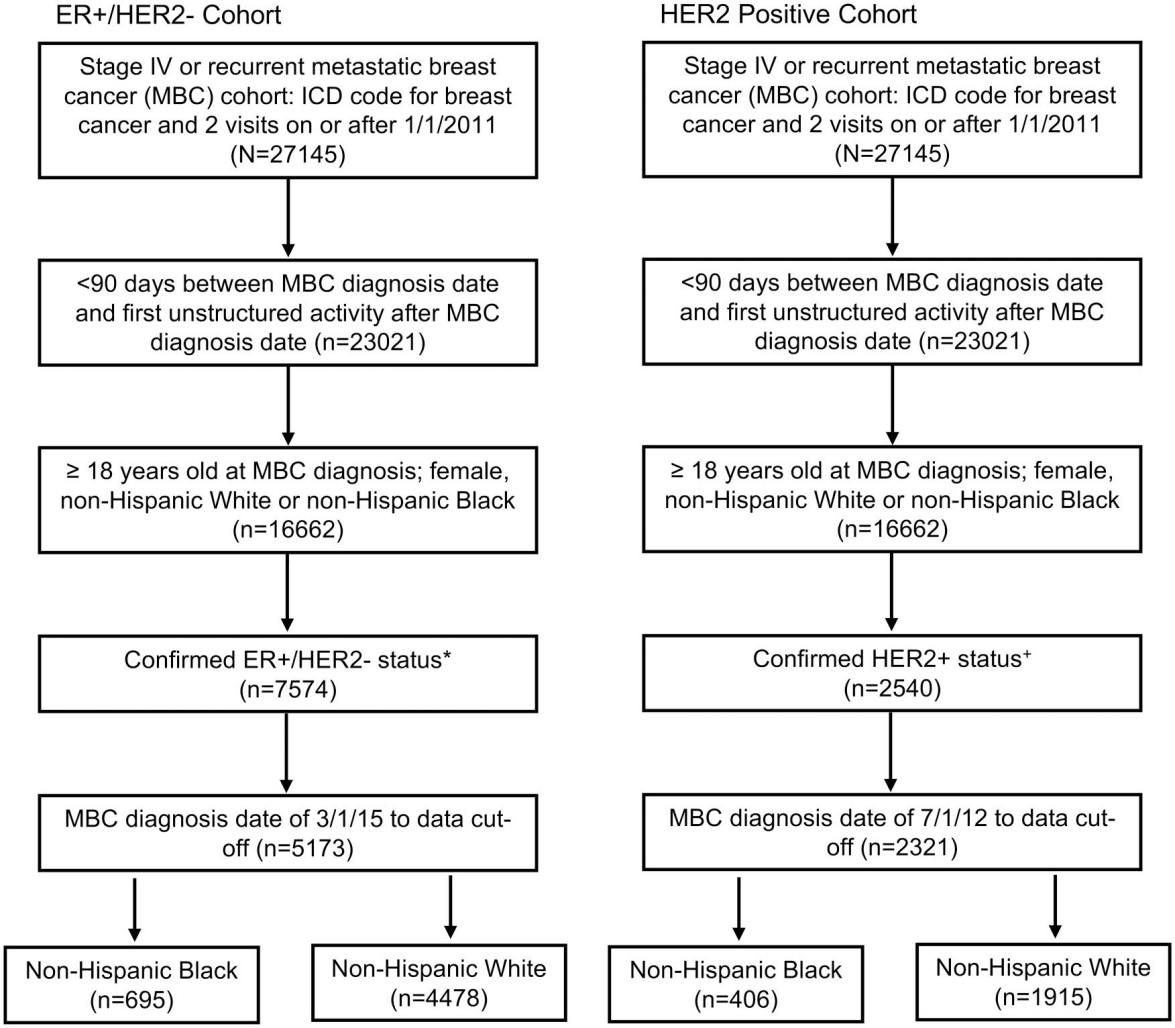
Patient attrition diagram. ^a^Confirmed ER+/HER2− status defined as any ER+ test before first-line MBC therapy start date; HER2− is defined as any HER2− test and the absence of a positive test before first-line MBC therapy date. ^b^Confirmed HER2+ status defined as any positive HER2 status before first-line MBC therapy start date. Abbreviations: ER+ = estrogen receptor positive; ICD = *International Classification of Diseases*; MBC = metastatic breast cancer.

Overall, 7494 patients from the EHR-derived database met inclusion criteria for this cohort analysis (Figure 1). The estrogen receptor–positive and HER2-negative metastatic breast cancer cohort consisted of 5173 patients: 695 non-Hispanic Black (13.4%) and 4478 non-Hispanic White (86.6%). The HER2-positive metastatic breast cancer cohort consisted of 2321 patients: 406 non-Hispanic Black (17.5%) and 1915 non-Hispanic White (82.5%). Baseline clinical and demographic characteristics are presented in Table 1. Median age at diagnosis for the estrogen receptor–positive and HER2-negative metastatic breast cancer cohort was lower in non-Hispanic Black compared with non-Hispanic White patients (median = 63, IQR = 53-71 years, vs median = 67, IQR = 58-75 years). We noted a similar trend in the HER2-positive metastatic breast cancer cohort with a median age at diagnosis of 59 (IQR = 50-68) years in non-Hispanic Black compared with 63 (IQR = 53-72) years in non-Hispanic White.

**Table 1.** Baseline characteristics of patients in respective cohorts.

Characteristics	ER+/HER2− metastatic breast cancer (n = 5173)		HER2+ metastatic breast cancer (n = 2321)	
	Non-Hispanic Black (n = 695)	Non-Hispanic White (n = 4478)	Non-Hispanic Black (n = 406)	Non-Hispanic White (n = 1915)
Age at metastatic breast cancer diagnosis, median (IQR), y^a^	63 (53-71)	67 (58-75)	59 (50-68)	63 (53-72)
Metastatic breast cancer type, No. (%)				
De novo	240 (35)	1294 (29)	173 (43)	783 (41)
Recurrent	453 (65)	3181 (71)	231 (57)	1129 (59)
Unknown	≤3 (<0.1)	≤3 (<0.1)	≤5 (<0.1)	≤5 (<0.1)
Site of metastatic disease, No. (%)				
Adrenal	3 (0.4)	34 (0.8)	≤5 (<0.1)	13 (0.7)
Bone	312 (45)	2317 (52)	143 (35)	676 (35)
Bone marrow	6 (0.9)	67 (1.5)	≤5 (<0.1)	11 (0.6)
Brain	14 (2)	87 (2)	17 (4)	120 (6)
Central nervous system site	4 (0.6)	25 (0.6)	≤5 (<0.1)	11 (0.6)
Distant lymph node	103 (15)	487 (11)	69 (17)	314 (16)
Kidney	0 (0)	≤1 (<0.1)	≤5 (<0.1)	≤5 (<0.1)
Liver	68 (9.8)	447 (10)	64 (16)	312 (16)
Lung	99 (14)	443 (9.9)	72 (18)	290 (15)
Ovary	4 (0.6)	19 (0.4)	≤5 (<0.1)	6 (0.3)
Pancreas	0 (0)	5 (0.1)	≤5 (<0.1)	≤5 (<0.1)
Peritoneum	8 (1.2)	55 (1.2)	2 (0.5)	≤5 (<0.1)
Pleura	39 (5.6)	200 (4.5)	16 (4)	51 (3)
Skin	12 (1.7)	96 (2.2)	≤5 (<0.1)	36 (2)
Soft tissue	11 (1.6)	65 (1.5)	≤5 (<0.1)	22 (1)
Spleen	0 (0)	8 (0.2)	≤5 (≤1.2)	≤5 (<0.1)
Thyroid	1 (0.1)	2 (<0.1)	≤5 (<0.1)	≤5 (<0.1)
Other	8 (1.2)	98 (2.2)	7 (2)	33 (2)
Unknown	3 (0.1)	22 (0.4)	≤5 (<0.1)	6 (0.3)
Eastern Cooperative Oncology Group performance status, No. (%)				
0	273 (46)	1704 (46)	161 (40)	699 (37)
1	215 (36)	1331 (36)	106 (25)	536 (28)
2	76 (13)	470 (13)	43 (11)	188 (10)
3 or 4	26 (4.4)	193 (5.2)	14 (3)	57 (3)
Unknown	105 (15)	780 (17)	82 (20)	435 (23)
Practice setting, No. (%)				
Academic	43 (6.2)	389 (8.7)	25 (6)	183 (10)
Community	652 (94)	4089 (91)	381 (94)	1732 (90)
Insurance type, No. (%)				
Private, commercial	313 (45)	2367 (53)	203 (50)	945 (49)
Medicaid, Medicare, other government	163 (23)	925 (21)	82 (20)	326 (17)
Uninsured, self-pay	219 (32)	1186 (26)	121 (30)	644 (34)
State Medicaid expansion at diagnosis, No. (%)				
Yes	327 (47)	3065 (68)	177 (44)	1280 (67)
No	368 (53)	1413 (32)	229 (56)	635 (33)
Proportion Black patients at practice, median (IQR)	0.19 (0.12-0.29)	0.09 (0.03-0.13)	0.20 (0.12-0.37)	0.11 (0.03-0.13)

Abbreviations: ER = estrogen receptor; IQR = interquartile range.

a Patients with a birth year of 1938 or earlier may have an adjusted birth year in Flatiron Health datasets because of patient de-identification requirements.

### CDK4/6 inhibitor utilization

**Figure 2.**
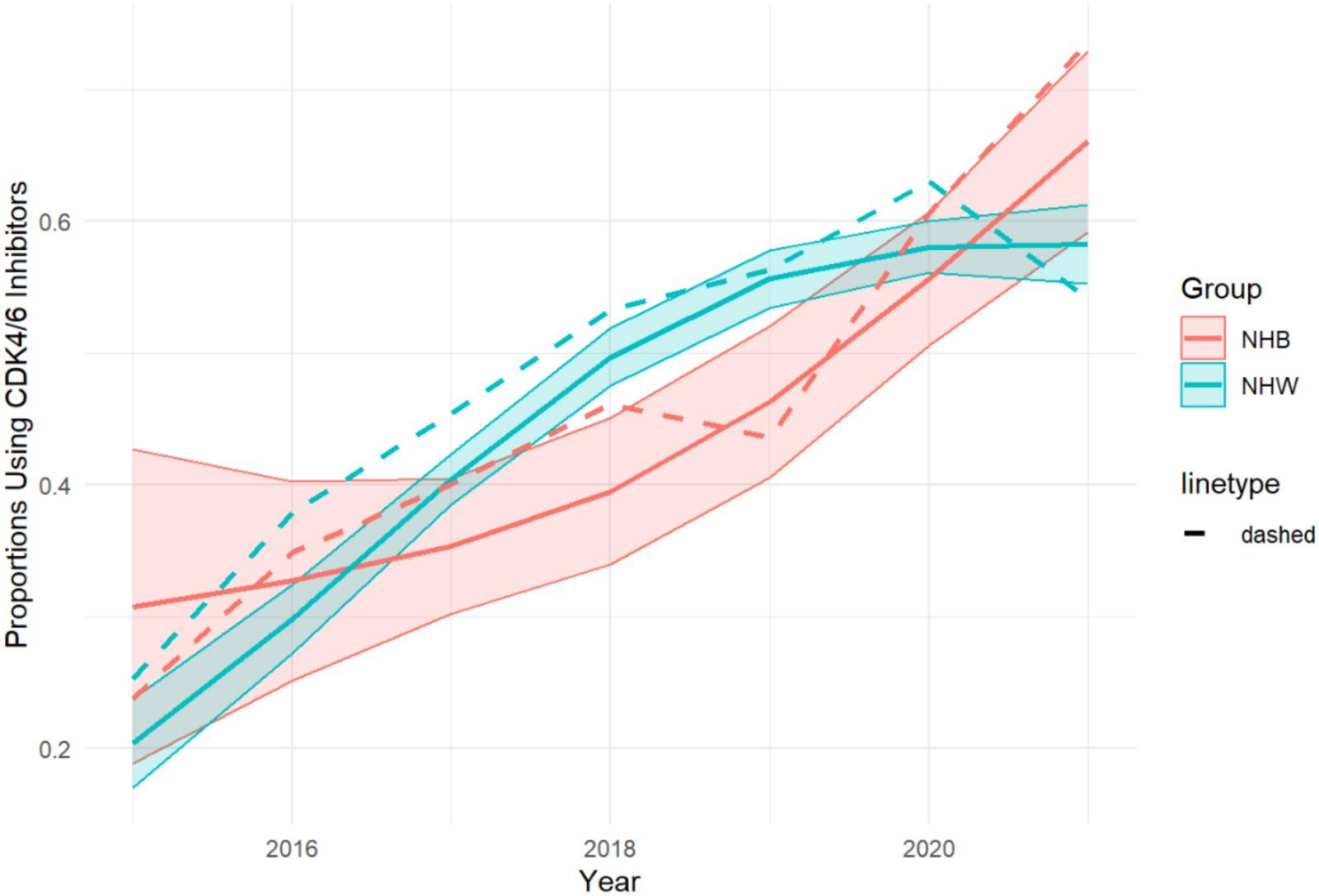
Unadjusted and adjusted time trends in proportions of eligible patients with metastatic breast cancer receiving first-line CDK4/6 inhibitors. **Dashed lines** represent unadjusted proportions (raw data). **Bold lines** and **shaded**  **areas** represent adjusted proportions and corresponding 95% confidence interval. Abbreviations: NHB = non-Hispanic Black; NHW = non-Hispanic White.

In 2015, the proportions of non-Hispanic White and non-Hispanic Black patients with estrogen receptor–positive and HER2-negative metastatic breast cancer receiving first-line CDK4/6 inhibitors were 23.5% (95% CI = 20.1% to 27.3%) and 20.6% (95% CI = 11.9% to 33.0%), which increased to 53.8% (95% CI = 48.6% to 59.0%) and 73.6% (95% CI = 61.7% to 83.0%) in 2021, respectively (Table 2, Figure 2). In the adjusted model there was a statistically significant increase in the proportion of non-Hispanic White patients with metastatic breast cancer (*P* = .009) and the proportion of non-Hispanic Black patients with metastatic breast cancer (*P* < .001) receiving CDK4/6 inhibitors over time (Figure 2). Additionally, there was a statistically significant difference in the temporal trends in utilization between non-Hispanic White and non-Hispanic Black patients with metastatic breast cancer (*P* = .009), with non-Hispanic Black race associated with lower CDK4/6 inhibitor utilization at most timepoints. Lower performance status, ECOG performance status 2-4 (OR = 0.84, 95% CI = 0.78 to 0.90; *P* < .001), was associated with lower odds of CDK4/6 inhibitor receipt compared with ECOG performance status 0. A larger proportion of non-Hispanic Black patients seen at a practice was also associated with statistically significantly lower odds of initiating CDK4/6 inhibitors (OR = 0.99, 95% CI = 0.98 to 0.99) per 10% increase in the proportion of Black patients at the practice (*P* < .001). Lastly, care at a community practice was associated with higher odds of receipt of CDK4/6 inhibitors (OR = 1.52, 95% CI = 1.22 to 1.90; *P* < .001) (Table S1).

**Table 2.** Unadjusted proportion and 95% confidence interval of eligible patients with metastatic breast cancer receiving first-line CDK 4/6 inhibitors.

Year	Non-Hispanic White			Non-Hispanic Black		
	Proportion, %	95% CI	No.	Proportion, %	95% CI	No.
2015	23.5	20.1 to 27.3	133	20.6	11.9 to 33.0	13
2016	37.1	33.6 to 40.7	273	34.0	25.2 to 43.9	36
2017	44.2	40.5 to 48.0	306	36.2	27.2 to 46.2	38
2018	51.0	47.4 to 54.6	390	41.9	33.0 to 51.4	49
2019	54.3	50.7 to 57.9	407	43.6	35.1 to 52.5	58
2020	61.7	57.7 to 65.5	375	59.6	49.2 to 69.2	59
2021	53.8	48.6 to 59.0	196	73.6	61.7 to 83.0	53

### Pertuzumab utilization

**Figure 3.**
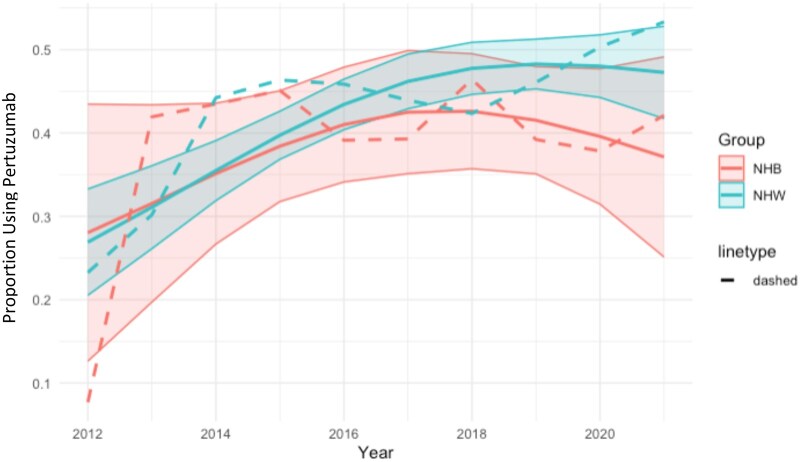
Unadjusted and adjusted temporal trends in the proportion of eligible patients initiating pertuzumab by race. **Dashed lines** represent unadjusted proportions (raw data). **Bold lines** and **shaded**  **areas** represent adjusted proportions and corresponding 95% confidence interval. Abbreviations: NHB = non-Hispanic Black; NHW = non-Hispanic White.

In 2012, the unadjusted proportion of non-Hispanic White and non-Hispanic Black patients with HER2-positive metastatic breast cancer who initiated first-line pertuzumab was 19.8% (95% CI = 12.3% to 30.0%) and 7.7% (95% CI = 0.4% to 37.9%), respectively, which increased to 52.2% (95% CI = 41.5% to 62.8%) and 42.1% (95% CI = 21.1% to 66.0%) in 2021 (Table 3). The proportions of non-Hispanic White patients with metastatic breast cancer (*P* < .001) and non-Hispanic Black patients (*P* < .001) utilizing first-line pertuzumab statistically significantly increased within groups; however, there was no statistically significant difference between the respective temporal trends for non-Hispanic White and non-Hispanic Black patients with metastatic breast cancer (*P* = .45) (Figure 3). ECOG performance status 2-4 compared with performance status 0 was associated with lower odds of utilizing pertuzumab (OR = 0.68, 95% CI = 0.55 to 0.84; *P* < .001) (Table S2).

**Table 3.** Unadjusted proportions and 95% confidence interval of eligible patients with metastatic breast cancer receiving first-line pertuzumab.

Year	Non-Hispanic White			Non-Hispanic Black		
	Proportion, %	95% CI	No.	Proportion, %	95% CI	No.
2012	19.8	12.3 to 30.0	86	7.7	0.4 to 37.9	13
2013	25.2	19.8 to 31.6	222	32.3	17.3 to 51.5	31
2014	39.9	33.3 to 46.9	208	43.5	29.2 to 58.8	46
2015	43.8	37.4 to 50.4	233	43.1	29.6 to 57.7	51
2016	45.0	38.7 to 51.5	242	37.0	23.6 to 52.5	46
2017	40.9	34.9 to 47.1	264	37.5	25.2 to 51.5	56
2018	41.4	34.9 to 48.2	222	41.1	28.4 to 54.9	56
2019	45.0	37.9 to 52.4	191	37.3	24.5 to 51.9	51
2020	49.0	41.0 to 57.1	157	32.4	18.6 to 49.9	37
2021	52.2	41.5 to 62.8	90	42.1	21.1 to 66.0	19

### Time to treatment initiation

Among the eligible cohort of women who received first-line CDK4/6 inhibitors within 90 days of metastatic breast cancer diagnosis, there was no statistically significant difference between the mean time to treatment initiation for non-Hispanic White compared with non-Hispanic Black women averaged over the period assessed (24.8 vs 26.2 days, respectively; *P* = .59). Mean time to treatment initiation decreased over time for non-Hispanic White and non-Hispanic Black patients initiating CDK4/6 inhibitors, representing shorter interval to treatment initiation. There was no statistically significant difference between the temporal trend for non-Hispanic White and non-Hispanic Black women (*P* = .28) (Figure S1). Similarly, among the eligible cohort who received first-line pertuzumab within 90 days of metastatic breast cancer diagnosis, there was no statistically significant difference between the mean time to treatment initiation for non-Hispanic White and non-Hispanic Black women averaged over the follow-up period (45.2 vs to 53.4 days, respectively; *P* = 0.23). Mean time to treatment initiation decreased over time for non-Hispanic White and non-Hispanic Black patients initiating pertuzumab; however, there was no statistically significant difference in temporal trends between non-Hispanic White and non-Hispanic Black women (*P* = .75) (Figure S2).

## Discussion

In this study utilizing an EHR-derived database, our analysis of patients with metastatic breast cancer eligible for CDK4/6 inhibitors and pertuzumab based on breast cancer receptor subtype, following FDA approval, showed underutilization of indicated first-line targeted therapies for HER2-positive metastatic breast cancer. Almost 50% of all eligible patients were not receiving guideline-recommended first-line pertuzumab within 90 days following diagnosis, critically limiting its therapeutic benefit. Moreover, non-Hispanic Black race was associated with lower CDK4/6 inhibitor utilization at all timepoints, except for 2021. Overall time to treatment initiation trends decreased over time following approval, and there were no racial differences in time to treatment initiation for either medicine.

The lower-than-expected rates of overall utilization reported in pertuzumab utilization are alarming, given its demonstrated survival benefit. Methodologically, the gross underutilization may be reflective of our analytic cohort restriction to patients initiating first-line therapy within 90 days of diagnosis. It is possible that a proportion of patients received these therapies more than 90 days after metastatic breast cancer diagnosis. However, our data show general decline in time to treatment initiation over time. Furthermore, prior analyses have shown treatment within 90 days as an important quality metric associated with survival, emphasizing the need for utilization and timeliness.^23^^,^^24^ Pertuzumab underutilization in the first-line metastatic breast cancer setting may partly reflect its prior use in the (neo)adjuvant setting, where it would not be indicated for patients who later present with recurrent metastatic disease after already receiving this therapy. However, this rationale is unlikely to account for the observed trends in CDK4/6 inhibitor use, as these agents were not approved for the adjuvant setting during the years covered in this analysis. Our findings align with other real-world analyses that show that although there is a general increasing trend of utilization of newer cancer therapies across multiple cancer types following drug approval,^26^^,^^27^ utilization rates for many novel anticancer medicines and diagnostic tests remain low several years after approval.^28^^,^^29^ Importantly, this is in contrast to an analysis of utilization of immune checkpoint inhibitors of programmed cell death 1 protein (anti-PD-L1 agents), which showed rapid uptake within 4 months following FDA approval for melanoma, non-small cell lung cancer, and renal cell carcinoma, with a cumulative uptake of 68.7% of eligible patients receiving these anti-PD-L1 agents following FDA approval.^30^ The anti-PD-L1 study may be unique in that it offered major survival gains for diseases that previously had poor prognosis (eg, BRAF wild-type unresectable metastatic melanoma).^31^ Our analysis, however, is reflective of a more general trend of the critical gap in the translation of evidence-based clinical guidelines into practice, which undermines the therapeutic benefit of these drugs and potentially untapped survival benefit. It also indicates a need to focus quality improvement and implementation science initiatives around adherence to guideline concordant cancer care.

It is important to note that despite their proven efficacy in the real-world setting, cost may be an important determinant of underutilization that we did not assess. Some cost-effectiveness analyses have shown that the addition of pertuzumab to docetaxel and trastuzumab for metastatic breast cancer may not be cost effective in several health-care systems, including the United States, which may influence provider prescription patterns for pertuzumab.^32^^,^^33^ Similar results have been demonstrated in cost-effectiveness analysis of CDK4/6 inhibitors in addition to aromatase inhibitors.^34^ Furthermore, emerging data suggest that CDK4/6 inhibitors may be more cost effective in the second-line metastatic breast cancer setting and is currently being investigated in the SONIA trial,^35^ which is a phase III trial comparing first-line vs second-line use of CDK4/6 inhibitors plus endocrine therapy in the Netherlands. Additionally, the duration of CDK4/6 inhibitor utilization was 24.6 months if given in the first-line setting compared with 8.1 months in the second-line setting with no difference in clinical benefit, suggesting that second-line CDK4/6 inhibitor utilization may be more cost-effective.^36^

We also noted slightly higher utilization of CDK4/6 inhibitors but persistent lower utilization in non-Hispanic Black race compared with non-Hispanic White except for 2021. This may be related to reimbursement policies for these medicines. Oral cancer medicines are usually classified as specialty drugs that carrysubstantial out-of-pocket costs that are due upfront at the time of pick-up at a pharmacy as opposed to out-of-pocket costs for IV drugs that are usually billed after medical services have been delivered.^37^ Furthermore, whereas out-of-pocket costs for IV medicines have remained fairly constant, there is an increasing trend in out-of-pocket costs for oral targeted medicines.^38^^,^^39^ Higher prescription drug pricing has the potential to affect initiation and adherence of these medicines with data showing that up to 41% of patients with copay between $100 and $500 and 67% of patients with co-pay more than $2000 did not purchase an approved oral cancer therapy prescription.^31^ Although our current analysis did not account for socioeconomic status or neighborhood deprivation, lower neighborhood-level of deprivation indices are associated with lower rates of cancer treatment initiation, which may partly explain the disparities in oral CDK4/6 inhibitor utilization.^40^^,^^41^

The trend in 2021 showed potential reversal of racial disparities in CDK4/6 inhibitor utilization. Recent studies suggest potential narrowing of racial disparities,^42^ however the data cutoff in 2021 is incomplete, and hence, more longitudinal data are needed to explore this trend and examine any potential impact of the COVID-19 pandemic. Of note, our analysis is consistent with prior publications that have shown a similar association between poor performance status and underutilization of cancer therapies compared with patients with good performance status.^43^ Additionally, our analysis showed that Medicaid expansion was associated with lower utilization of CDK4/6 inhibitors, which is in contrast to studies that have shown improved cancer survival, earlier stage at diagnosis, and reduced treatment disparities.^44^^,^^45^ However, these studies have focused on broader treatment access and timing outcomes, not specific novel cancer therapies. Finally, cancer practices with a higher proportion of Black patients had lower likelihood of CDK4/6 inhibitor utilization. Prior studies have demonstrated that care for minority patients tends to be clustered into relatively few hospitals with disproportionately high minority patient populations.^46^ Furthermore, hospitals that deliver care to majority minority populations tend to be relatively poorer in financial assets and have worse health outcome indicators.^25^^,^^47^

Our study has some limitations: First, a majority of patients included in this cohort were seen at community practices, and these results may not be generalizable to academic practice settings. However, these results are reflective of where most Americans receive cancer care. Additionally lower utilization rates may reflect the restriction to a 90-day window for treatment initiation in our analysis. Furthermore, potential mediators of therapy utilization, for instance presentation with visceral crisis or comorbidities, were not available in the database and may influence metastatic breast cancer therapy choice. Another limitation is that this study did not analyze other racial and ethnic groups as these numbers were too small for inferential analysis. Lastly, detailed socioeconomic factors and neighborhood-level social determinants of health are important covariates that were not captured in our analysis because these were not available in the EHR-derived database at the time of data requisition.

In conclusion, given the rapidly evolving landscape of new cancer therapeutics, it is important to understand how evidence-based guidelines are translated into clinical practice. The consistent reports of underutilization of these medicines with proven efficacy suggest that there is untapped potential to further improve on cancer outcomes. This analysis of real-world data is important for calling into focus quality improvement initiatives designed to identify determinants and multilevel interventions for improving adherence to guideline concordant cancer guidelines. Embedded in these initiatives should be an equity lens that ensures that resources are tailored to mitigate barriers that exacerbate disparities in cancer care access. Future studies should explore how these utilization trends are mediated by social economic status and social determinants of health, as well as build on some of our important findings such as associations between Medicaid expansion under the ACA and access to specific cancer-targeted therapies.

In this cohort analysis of women with metastatic breast cancer, we observed that approximately half of eligible patients from both racial groups did not receive pertuzumab, limiting its therapeutic benefit. Non-Hispanic Black were less likely to receive CDK4/6 inhibitors compared with non-Hispanic White. Further research is needed to understand mediators of underutilization of newer therapies.

## Supplementary Material

pkag043_Supplementary_Data

## Data Availability

Data are available upon reasonable request. The data that support the findings of this study originated from Flatiron Health. Requests for data sharing by license or by permission for the specific purpose of replicating results in this manuscript can be submitted to publicationsdataaccess@flatiron.com.

## References

[1] Smigal C , Jemal A, Ward E, et al Trends in breast cancer by race and ethnicity: update 2006. CA Cancer J Clin. 2006;56:168-183. 10.3322/canjclin.56.3.168 16737949

[2] Silber JH , Rosenbaum PR, Clark AS, et al Characteristics associated with differences in survival among black and white women with breast cancer. JAMA. 2013;310:389-397. 10.1001/jama.2013.8272 23917289

[3] Wang F , Zheng W, Bailey CE, Mayer IA, Pietenpol JA, Shu XO. Racial/Ethnic disparities in all-cause mortality among patients diagnosed with triple-negative breast cancer. Cancer Res. 2021;81:1163-1170. 10.1158/0008-5472.CAN-20-3094 33272926 PMC10571320

[4] Hill DA , Prossnitz ER, Royce M, Nibbe A. Temporal trends in breast cancer survival by race and ethnicity: a population-based cohort study. PLoS One. 2019;14:e0224064. 10.1371/journal.pone.0224064 31647839 PMC6812853

[5] Tao L , Gomez SL, Keegan THM, Kurian AW, Clarke CA. Breast cancer mortality in African-American and non-hispanic white women by molecular subtype and stage at diagnosis: a population-based study. Cancer Epidemiol Biomarkers Prev. 2015;24:1039-1045. 10.1158/1055-9965.EPI-15-0243 25969506 PMC4490947

[6] Reeder-Hayes KE , Mayer SE, Olshan AF, et al Race and delays in breast cancer treatment across the care continuum in the Carolina Breast Cancer Study. Cancer. 2019;125:3985-3992. 10.1002/cncr.32378 31398265 PMC6819218

[7] Emerson MA , Golightly YM, Aiello AE, et al Breast cancer treatment delays by socioeconomic and health care access latent classes in Black and White women. Cancer. 2020;126:4957-4966. 10.1002/cncr.33121 32954493 PMC7789230

[8] George P , Chandwani S, Gabel M, et al Diagnosis and surgical delays in African American and white women with early-stage breast cancer. J Womens Health 2002. 2015;24:209-217. 10.1089/jwh.2014.4773 PMC4442576 25650628

[9] Cronin KA, Richardson LC, Henley SJ, et al. Vital Signs: Racial Disparities in Breast Cancer Severity–United States, 2005–2009. Accessed March 30, 2024. https://www.cdc.gov/mmwr/preview/mmwrhtml/mm6145a5.htm

[10] Vaz-Luis I , Lin NU, Keating NL, et al Treatment of early-stage human epidermal growth factor 2-positive cancers among medicare enrollees: age and race strongly associated with non-use of trastuzumab. Breast Cancer Res Treat. 2016;159:151-162. 10.1007/s10549-016-3927-4 27484879

[11] Chen L , Li CI. Racial disparities in breast cancer diagnosis and treatment by hormone receptor and HER2 status. Cancer Epidemiol Biomarkers Prev. 2015;24:1666-1672. 10.1158/1055-9965.EPI-15-0293 26464428 PMC4633380

[12] Cristofanilli M , Turner NC, Bondarenko I, et al Fulvestrant plus palbociclib versus fulvestrant plus placebo for treatment of hormone-receptor-positive, HER2-negative metastatic breast cancer that progressed on previous endocrine therapy (PALOMA-3): final analysis of the multicentre, double-blind, phase 3 randomised controlled trial. Lancet Oncol. 2016;17:425-439. 10.1016/S1470-2045(15)00613-0 26947331

[13] Goetz MP , Toi M, Campone M, et al MONARCH 3: Abemaciclib as initial therapy for advanced breast cancer. J Clin Oncol. 2017;35:3638-3646. 10.1200/JCO.2017.75.6155 28968163

[14] O’Shaughnessy J , Petrakova K, Sonke GS, et al Ribociclib plus letrozole versus letrozole alone in patients with de novo HR+, HER2- advanced breast cancer in the randomized MONALEESA-2 trial. Breast Cancer Res Treat. 2018;168:127-134. 10.1007/s10549-017-4518-8 29164421 PMC5847028

[15] Swain SM , Kim SB, Cortés J, et al Pertuzumab, trastuzumab, and docetaxel for HER2-positive metastatic breast cancer (CLEOPATRA study): overall survival results from a randomised, double-blind, placebo-controlled, phase 3 study. Lancet Oncol. 2013;14:461-471. 10.1016/S1470-2045(13)70130-X 23602601 PMC4076842

[16] Swain SM , Baselga J, Kim SB, et al; CLEOPATRA Study Group. Pertuzumab, trastuzumab, and docetaxel in HER2-positive metastatic breast cancer. N Engl J Med. 2015;372:724-734. 10.1056/NEJMoa1413513 25693012 PMC5584549

[17] Finn RS , Martin M, Rugo HS, et al Palbociclib and Letrozole in advanced breast cancer. N Engl J Med. 2016;375:1925-1936. 10.1056/NEJMoa1607303 27959613

[18] Hortobagyi GN , Stemmer SM, Burris HA, et al Overall survival with Ribociclib plus Letrozole in advanced breast cancer. N Engl J Med. 2022;386:942-950. 10.1056/NEJMoa2114663 35263519

[19] Johnston S , Martin M, Di Leo A, et al MONARCH 3 final PFS: a randomized study of Abemaciclib as initial therapy for advanced breast cancer. NPJ Breast Cancer. 2019;5:5. 10.1038/s41523-018-0097-z 30675515 PMC6336880

[20] Benjamin L , Buthion V, Vidal-Trécan G, Briot P. Impact of the healthcare payment system on patient access to oral anticancer drugs: an illustration from the French and United States contexts. BMC Health Serv Res. 2014;14:274. 10.1186/1472-6963-14-274 24950778 PMC4082413

[21] Ma X , Long L, Moon S, Adamson BJS, Baxi SS. Comparison of Population Characteristics in Real-World Clinical Oncology Databases in the US: Flatiron Health, SEER, and NPCR. 2023. 10.1101/2020.03.16.20037143

[22] Birnbaum B , Nussbaum N, Seidl-Rathkopf K, et al Model-assisted cohort selection with bias analysis for generating large-scale cohorts from the EHR for oncology research. Published online January 13, 2020. 10.48550/arXiv.2001.09765

[23] Richards MA , Westcombe AM, Love SB, Littlejohns P, Ramirez AJ. Influence of delay on survival in patients with breast cancer: a systematic review. Lancet Lond Engl. 1999;353:1119-1126. 10.1016/s0140-6736(99)02143-1 10209974

[24] McLaughlin JM , Anderson RT, Ferketich AK, Seiber EE, Balkrishnan R, Paskett ED. Effect on survival of longer intervals between confirmed diagnosis and treatment initiation among low-income women with breast cancer. J Clin Oncol. 2012;30:4493-4500. 10.1200/JCO.2012.39.7695 23169521 PMC3518728

[25] Himmelstein G , Himmelstein KEW. Inequality set in concrete: physical resources available for care at hospitals serving people of color and other U.S. hospitals. Int J Health Serv Plan Adm Eval. 2020;50:363-370. 10.1177/0020731420937632 32611234

[26] Doshi JA , Jahnke J, Raman S, et al Treatment utilization patterns of newly initiated oral anticancer agents in a national sample of Medicare beneficiaries. J Manag Care Spec Pharm. 2021;27:1457-1468. 10.18553/jmcp.2021.27.10.1457 34595957 PMC10391122

[27] Parikh RB , Min EJ, Wileyto EP, et al Uptake and survival outcomes following immune checkpoint inhibitor therapy among trial-ineligible patients with advanced solid cancers. JAMA Oncol. 2021;7:1843-1850. 10.1001/jamaoncol.2021.4971 34734979 PMC8569600

[28] Lin HM , Wu Y, Yin Y, et al Real-world ALK testing trends in patients with advanced non–small-cell lung cancer in the United States. Clin Lung Cancer. 2023;24:e39-e49. 10.1016/j.cllc.2022.09.010 36376172

[29] Rodriguez-Quintero JH , Kamel MK, Jindani R, et al Is underutilization of adjuvant therapy in resected non-small-cell lung cancer associated with socioeconomic disparities? Eur J Cardio-Thorac Surg. 2023;64:ezad383. 10.1093/ejcts/ezad383 PMC11007729 37952179

[30] O’Connor JM , Fessele KL, Steiner J, et al Speed of adoption of immune checkpoint inhibitors of programmed cell death 1 protein and comparison of patient ages in clinical practice vs pivotal clinical trials. JAMA Oncol. 2018;4:e180798. 10.1001/jamaoncol.2018.0798 29800974 PMC6143052

[31] Beaver JA , Theoret MR, Mushti S, et al FDA approval of Nivolumab for the first-line treatment of patients with BRAFV600 wild-type unresectable or metastatic melanoma. Clin Cancer Res. 2017;23:3479-3483. 10.1158/1078-0432.CCR-16-0714 28073844

[32] Dai WF , Beca JM, Nagamuthu C, et al Cost-effectiveness analysis of Pertuzumab with Trastuzumab in patients with metastatic breast cancer. JAMA Oncol. 2022;8:597-606. 10.1001/jamaoncol.2021.8049 35201264 PMC8874900

[33] Durkee BY , Qian Y, Pollom EL, et al Cost-effectiveness of Pertuzumab in human epidermal growth factor receptor 2-positive metastatic breast cancer. J Clin Oncol. 2016;34:902-909. 10.1200/JCO.2015.62.9105 26351332 PMC5070553

[34] Masurkar PP , Damgacioglu H, Deshmukh AA, Trivedi MV. Cost effectiveness of CDK4/6 inhibitors in the first-line treatment of HR+/HER2- metastatic breast cancer in postmenopausal women in the USA. PharmacoEconomics. 2023;41:709-718. 10.1007/s40273-023-01245-y 36920662

[35] van Ommen-Nijhof A , Konings IR, van Zeijl CJJ, et al; SONIA study steering committee. Selecting the optimal position of CDK4/6 inhibitors in hormone receptor-positive advanced breast cancer - the SONIA study: study protocol for a randomized controlled trial. BMC Cancer. 2018;18:1146. 10.1186/s12885-018-4978-1 30458732 PMC6247672

[36] Sonke GS , Van Ommen- Nijhof A, Wortelboer N, et al Primary outcome analysis of the phase 3 SONIA trial (BOOG 2017-03) on selecting the optimal position of cyclin-dependent kinases 4 and 6 (CDK4/6) inhibitors for patients with hormone receptor-positive (HR+), HER2-negative (HER2-) advanced breast cancer (ABC). JCO. 2023;41:LBA1000-LBA1000. 10.1200/JCO.2023.41.17_suppl.LBA1000

[37] Doshi JA , Li P, Huo H, Pettit AR, Armstrong KA. Association of patient out-of-pocket costs with prescription abandonment and delay in fills of novel oral anticancer agents. J Clin Oncol. 2018;36:476-482. 10.1200/JCO.2017.74.5091 29261440

[38] Shih YCT , Smieliauskas F, Geynisman DM, Kelly RJ, Smith TJ. Trends in the cost and use of targeted cancer therapies for the privately insured Nonelderly: 2001 to 2011. J Clin Oncol. 2015;33:2190-2196. 10.1200/JCO.2014.58.2320 25987701 PMC4477789

[39] Shih YCT , Xu Y, Liu L, Smieliauskas F. Rising prices of targeted oral anticancer medications and associated financial burden on Medicare beneficiaries. J Clin Oncol. 2017;35:2482-2489. 10.1200/JCO.2017.72.3742 28471711 PMC5536165

[40] Guadamuz JS , Wang X, Ryals CA, et al Socioeconomic status and inequities in treatment initiation and survival among patients with cancer, 2011-2022. JNCI Cancer Spectr. 2023;7:pkad058. 10.1093/jncics/pkad058 37707536 PMC10582690

[41] Ren JX , Gong Y, Ling H, Hu X, Shao ZM. Racial/ethnic differences in the outcomes of patients with metastatic breast cancer: contributions of demographic, socioeconomic, tumor and metastatic characteristics. Breast Cancer Res Treat. 2019;173:225-237. 10.1007/s10549-018-4956-y 30293212 PMC6394580

[42] Krishnamurthy S , Jazowski SA, Roberson ML, et al Racial and ethnic disparities in receipt of ERBB2-targeted therapy for breast cancer, 2010-2020. JAMA Netw Open. 2025;8:e258086. 10.1001/jamanetworkopen.2025.8086 40310643 PMC12046428

[43] Sussell J , Boudreau D, Schuldt R, et al Treatment patterns and unmet need for patients with advanced non-small cell lung cancer and poor performance status: a real-world evidence study. J Clin Oncol. 2023;41:9077-9077.

[44] Akinyemi O , Oyebanji O, Fasokun M, et al Medicaid expansion and overall mortality among women with breast cancer. JAMA Netw Open. 2026;9:e2554512. 10.1001/jamanetworkopen.2025.54512. 41591779 PMC12848628

[45] Han X , Zhao J, Yabroff KR, Johnson CJ, Jemal A. Association between Medicaid expansion under the affordable care act and survival among newly diagnosed cancer patients. J Natl Cancer Inst. 2022;114:1176-1185. 10.1093/jnci/djac077. 35583373 PMC9360456

[46] Bach PB , Pham HH, Schrag D, Tate RC, Hargraves JL. Primary care physicians who treat blacks and whites. N Engl J Med. 2004;351:575-584. 10.1056/NEJMsa040609 15295050

[47] Aggarwal R , Hammond JG, Joynt Maddox KE, Yeh RW, Wadhera RK. Association between the proportion of black patients cared for at hospitals and financial penalties under value-based payment programs. JAMA. 2021;325:1219-1221. 10.1001/jama.2021.0026 33755063 PMC7988363

