# Supplementary material for: Racial disparities and utilization trends of first-line targeted therapies for metastatic breast cancer
Source: JNCI Cancer Spectr. 2026 Apr 24;10(3):pkag043. doi: 10.1093/jncics/pkag043 (PMC13265379; doi:10.1093/jncics/pkag043)
Supplement: pkag043_Supplementary_Data [file pkag043_supplementary_data.zip › Flatiron_MBCDisparities_JNCICS_Suppl_Materials_021026.docx]

**SUPPLEMENTARY MATERIALS**

Table of Contents

[Table S1: Odds ratios and 95% CI from logistic regression model for receipt of CDK4/6 inhibitors 2](#_Toc221602761)

[Table S2: Odds ratios and 95% CI from logistic regression model for receipt of Pertuzumab in the first-line metastatic breast cancer setting 3](#_Toc221602762)

[Figure S1: Unadjusted and adjusted model of predicted time to treatment initiation (TTI) of CDK4/6 inhibitors among NHB and NHW 4](#_Toc221602763)

[Figure S2: Unadjusted and adjusted model of predicted time to treatment initiation (TTI) of pertuzumab among NHW and NHB trends 5](#_Toc221602764)

# Table S1: Odds ratios and 95% CI from logistic regression model for receipt of CDK4/6 inhibitors

| Covariates | Odds Ratio | 95% CI | P value |
| --- | --- | --- | --- |
| Age* (years) | 1.00 | 0.99-1.00 | 0.245 |
|  |  |  |  |
| ECOG functional status |  |  |  |
| ECOG 0 | Ref |  |  |
| ECOG 1 | 0.84 | 0.78-0.90 | 0.002 |
| ECOG 2-4 | 0.74 | 0.66-0.83 | <0.001 |
|  |  |  |  |
| Insurance |  |  |  |
| Private/commercial | Ref |  |  |
| Medicare/Medicaid/ other govt | 0.96 | 0.85-1.09 | 0.53 |
| Uninsured/self-pay | 1.04 | 0.93-1.16 | 0.49 |
|  |  |  |  |
| No Medicaid expansion | Ref |  |  |
| Medicaid expansion | 0.78 | 0.68-0.90 | 0.002 |
|  |  |  |  |
| Proportion of black patients in practice* | 0.99 | 0.98-0.99 | <0.001 |
|  |  |  |  |
| Practice type |  |  |  |
| Academic | Ref |  |  |
| Community | 1.52 | 1.22-1.90 | <0.001 |
| *Continuous variable (per 10% increase in proportion of black patients) | | | |

# Table S2: Odds ratios and 95% CI from logistic regression model for receipt of Pertuzumab in the first-line metastatic breast cancer setting

| Covariates | Odds Ratio | 95% CI | P value |
| --- | --- | --- | --- |
| Age* (years) | 0.97 | 0.97-0.98 | <0.001 |
| ECOG functional status |  |  |  |
| ECOG 0 | Ref |  |  |
| ECOG 1 | 1.01 | 0.79-0.94 | 0.94 |
| ECOG 2-4 | 0.68 | 0.55-0.84 | <0.001 |
| Insurance |  |  |  |
| Private/commercial | Ref |  |  |
| Medicare/Medicaid/ other govt | 0.98 | 0.77-1.26 | 0.89 |
| Uninsured/self-pay | 1.04 | 0.85-1.26 | 0.73 |
|  |  |  |  |
| No Medicaid expansion | Ref |  |  |
| Medicaid expansion | 0.93 | 0.76-1.15 | 0.51 |
|  |  |  |  |
| Proportion of black patients in practice* | 0.95 | 0.87-1.03 | 0.27 |
|  |  |  |  |
| Practice type |  |  |  |
| Academic | Ref |  |  |
| Community | 0.89 | 0.66-1.22 | 0.47 |
| *Continuous variable (per 10% increase in proportion of black patients) | | | |

# Figure S1: Unadjusted and adjusted model of predicted time to treatment initiation (TTI) of CDK4/6 inhibitors among NHB and NHW


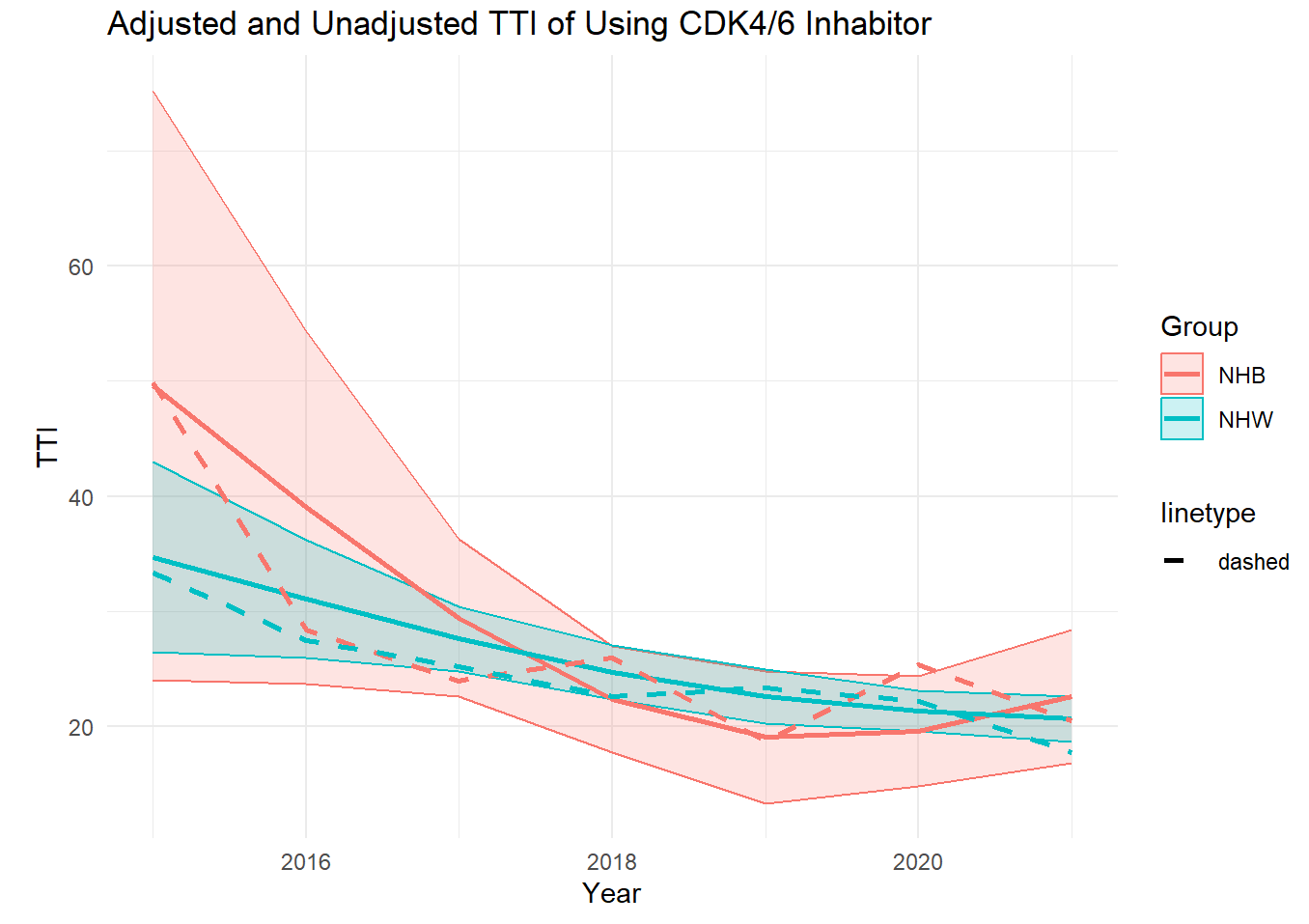


*Dashed lines represent unadjusted proportions (raw data). Bold lines and shaded areas represent adjusted proportions and corresponding 95% CI.

# Figure S2: Unadjusted and adjusted model of predicted time to treatment initiation (TTI) of pertuzumab among NHW and NHB trends


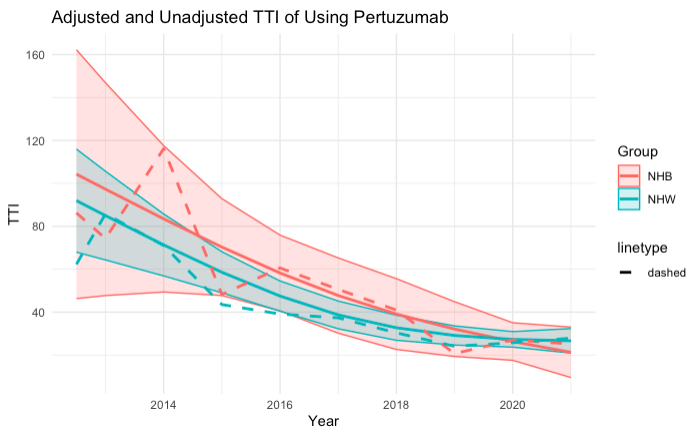


*Dashed lines represent unadjusted proportions (raw data). Bold lines and shaded areas represent adjusted proportions and corresponding 95% CI.
